# Publish/Subscribe Method for Real-Time Data Processing in Massive IoT Leveraging Blockchain for Secured Storage

**DOI:** 10.3390/s23249692

**Published:** 2023-12-08

**Authors:** Mohammadhossein Ataei, Ali Eghmazi, Ali Shakerian, Rene Landry, Guy Chevrette

**Affiliations:** 1 Department of Electrical Engineering, École de Technologie Supérieure, Montréal, QC H3C 1K3, Canada; ali.eghmazi@lassena.etsmtl.ca (A.E.); ali.shakerian@lassena.etsmtl.ca (A.S.); renejr.landry@etsmtl.ca (R.L.J.); 2 Corporate Office of iMETRIK Global Inc., Montreal, QC J4P 2K7, Canada; guy.chevrette@imetriklabs.com

**Keywords:** Massive Internet of Things (MIoT), publish/subscribe methodology, real-time data processing, Apache Kafka, Apache Druid, latency, Hyperledger Fabric, storage security

## Abstract

In the Internet of Things (IoT) era, the surge in Machine-Type Devices (MTDs) has introduced Massive IoT (MIoT), opening new horizons in the world of connected devices. However, such proliferation presents challenges, especially in storing and analyzing massive, heterogeneous data streams in real time. In order to manage Massive IoT data streams, we utilize analytical database software such as Apache Druid version 28.0.0 that excels in real-time data processing. Our approach relies on a publish/subscribe mechanism, where device-generated data are relayed to a dedicated broker, effectively functioning as a separate server. This broker enables any application to subscribe to the dataset, promoting a dynamic and responsive data ecosystem. At the core of our data transmission infrastructure lies Apache Kafka version 3.6.1, renowned for its exceptional data flow management performance. Kafka efficiently bridges the gap between MIoT sensors and brokers, enabling parallel clusters of brokers that lead to more scalability. In our pursuit of uninterrupted connectivity, we incorporate a fail-safe mechanism with two Software-Defined Radios (SDR) called Nutaq PicoLTE Release 1.5 within our model. This strategic redundancy enhances data transmission availability, safeguarding against connectivity disruptions. Furthermore, to enhance the data repository security, we utilize blockchain technology, specifically Hyperledger Fabric, known for its high-performance attributes, ensuring data integrity, immutability, and security. Our latency results demonstrate that our platform effectively reduces latency for 100,000 devices, qualifying as an MIoT, to less than 25 milliseconds. Furthermore, our findings on blockchain performance underscore our model as a secure platform, achieving over 800 Transactions Per Second in a dataset comprising 14,000 transactions, thereby demonstrating its high efficiency.

## 1. Introduction

The Internet of Things (IoT) revolutionizes our interaction with the surrounding objects, forming a network of diverse devices such as computers, sensors, intelligent gadgets, and smartphones. These devices are interconnected through various technologies to access the internet [1]. In upcoming years, through sixth-generation (6G) networks, the capacity to facilitate interactions among billions of interconnected devices and applications, all at remarkably elevated data speeds, emerges as a notable prospect which leads us to the Massive IoT (MIoT) concept [2]. These facts give rise to demanding attention, including rapid real-time data processing, scalability, storage security, availability, and latency. Therefore, the impetus to create this model arose from the requirement for fast information exchange and processing among IoT components, underpinned by a scalable, secure, and available framework with minimal latency.

IoT-based frameworks require key characteristics to function effectively. These include (i) connectivity among devices to enable collective intelligence, (ii) accurate and timely sensing for detecting environmental changes, (iii) intelligent analysis of gathered data with minimal latency to extract meaningful insights, (iv) dynamic adaptability to accommodate evolving systems, (v) scalability to handle increasing device and data volumes, (vi) ability to manage heterogeneity among devices and data types, (vii) and robust security measures to protect against cyber threats and data breaches. These attributes collectively shape a reliable and efficient IoT ecosystem [3]. According to the aforementioned characteristics, a framework that can satisfy requirements is necessary for supporting all the features [4].

The data generated by IoT sensors can be managed within a centralized environment such as a cloud. To effectively manage massive data from intelligent devices, it is necessary for the data to be efficiently and flexibly transmitted from the devices to the cloud [5]. IoT encompasses energy-efficient devices, often characterized by limited processing power, computation capacity, and battery capacity [6]. The publish/subscribe concept, offering event-triggered, asynchronous communication between publishers and subscribers, is well suited for extensive, distributed IoT services with limited energy, due to its thorough separation of publishers and subscribers across space, time, and synchronization [7].

In the domain of Massive IoT, numerous challenges require careful consideration for successful implementation. Foremost among these challenges is efficiently processing massive and heterogeneous data in real time with minimal latency. Simultaneously, ensuring network availability and establishing a secure, immutable data storage mechanisms remain paramount. Our research endeavors revolve around the development and validation of a novel approach, harnessing the power of a publish/subscribe method for real-time data processing in the realm of MIoT, while leveraging blockchain technology for the secure storage of data.

The core contributions of this research paper are as follows:

1. We introduce a comprehensive architectural framework, rooted in the publish/subscribe methodology, tailored for real-time data processing within the expansive domain of MIoT.
2. To tackle the intricacies of real-time data processing, we employ the robust capabilities of Apache Kafka version 3.6.1 and Apache Druid version 28.0.0.
3. Recognizing the significance of data storage security and immutability, we incorporate Hyperledger Fabric, a cutting-edge blockchain technology, to fortify our system’s data storage capabilities.
4. In pursuit of network availability and efficiency, we employed two Software-Defined Radios (SDRs) based on LTE as our “Network-in-a-box” through two distinct implementations, showcasing the potential for robust network connectivity.
5. Furthermore, we substantiate the effectiveness of our proposed model through a series of meticulously designed experiments. These experiments are aimed at quantifying latency and assessing the overall performance of our blockchain-integrated solution.

In doing so, our research not only offers a comprehensive architectural framework but also provides empirical evidence of its practicality and efficiency, thus contributing substantively to the field of MIoT.

### Related Work

The authors in [8] conducted a comprehensive state-of-the-art review, shedding light on how the microservice-based architecture is leveraged to enhance non-functional characteristics, specifically reliability and availability, in IoT ecosystems. Their pioneering work outlines several critical challenges addressed by MAs, including IoT device integration, heterogeneity, interoperability, fault tolerance, scalability, and system deployment and configuration. Moreover, they provide insights into the techniques proposed for handling non-functional requirements (NFRs) within MAs for IoT systems. Notably, for improving availability, reactive architecture, circuit breaker patterns, orchestration, and machine learning have emerged as prominent techniques, while messaging protocols have been favored for addressing interoperability challenges. Furthermore, for scalability, orchestration and load balancing techniques have been explored. However, they highlight that MAs have not been universally explored across all IoT domains, and most notably, another study identified a critical gap in the literature concerning end-to-end availability and reliability, emphasizing that this surpasses the mere availability of individual components within a system [9].

In [10], the proposed hybrid centralized and blockchain-based architecture addresses critical challenges encountered by resource-constrained IoT devices. The author believes that traditional cryptographic approaches have been extensively explored but are ill suited for the resource limitations of IoT devices, a concern that aligns with the findings of the study in [11]. The incorporation of blockchain technology for IoT authentication, as introduced in that study, has been a growing area of interest in recent years. However, that study’s emphasis on minimizing computational costs and addressing real-time requirements distinguishes it from existing blockchain-based approaches that often incur significant overhead. The novel hybrid architecture proposed herein, combining centralized and blockchain-based elements, represents a promising direction for efficiently bolstering IoT security while mitigating the resource and scalability challenges faced by IoT systems.

In the landscape of publish–subscribe systems, the article in [12] sheds light on the critical design features and performance metrics for several open-source systems such as Apache Kafka and RabbitMQ. The present work contributes by offering a rigorous evaluation of seven open-source systems, establishing common criteria for comparison, and providing insights into functionality and performance under real-world conditions. However, this study needs to further explore alternative systems like ActiveMQ, Apache Pulsar, ZeroMQ, Redis, and others, which would contribute to a more comprehensive understanding of publish–subscribe systems’ capabilities and limitations.

The author in [13] in the realm of fog computing contributes a valuable lightweight authentication scheme tailored for resource-constrained IoT devices and fog gateways. On the other hand, pre-shared key (PSK) authentication methods have been proposed as a solution for low-resource devices, although they have faced questions about their security robustness [14]. That study introduced an innovative approach by combining the Elliptic Curve Diffie–Hellman Ephemeral (ECDHE) key exchange algorithm with PSK authentication within a Message Queuing Telemetry Transport (MQTT) publish–subscribe framework in the context of distributed fog computing. However, that study’s focus on ECDHE-PSK authentication within the MQTT publish–subscribe architecture may limit its applicability to other fog computing and IoT contexts.

The article by [15] proposes the RTID framework by utilizing Apache Spark for efficient data processing, RESTful API, and OAuth 2.0 for secure access management. In the realm of real-time IoT data processing, prior research has delved into addressing the multifaceted challenges associated with efficiently managing massive data [16]. Traditional approaches, including 6LoWPAN [17] and IoT with Cloud system (CCS) [18], have been fundamental in shaping the IoT landscape but have faced limitations in terms of flexibility and data management efficiency. Although it contributes to enhancing real-time Massive IoT data processing, more comprehensive comparative studies with existing frameworks are necessary to firmly establish the RTID framework’s suitability and feasibility in practical deployment scenarios.

Contrary to previous efforts in the field of data processing in IoT, which often tackled individual challenges using various methods and architectures like publish/subscribe and microservice-based approaches, some of which did not consider the big scale in MIoT, our research takes a holistic approach. Our primary objective is to present a comprehensive framework that integrates all these challenges into a unified architecture. This paper aims to provide an intricate overview of real-time data processing in MIoT and to address these formidable challenges collectively.

The remainder of this paper unfolds as follows: in Section 2, we firstly present our pioneering proposed architecture—a comprehensive and robust publish/subscribe architecture designed to handle real-time data processing in the MIoT ecosystem. Moreover, each layer of this architecture and the data flow are detailed, offering a comprehensive understanding of its inner workings. Finally, we delve into the mechanics of our chosen publish/subscribe methodology, employing Apache Kafka and Druid, elucidating how these components synergize to facilitate efficient data processing. Section 3 explores experimental analysis, illuminating both the capabilities and limitations of our model. Section 4 initiates a thoughtful discussion, while Section 5 highlights a few key conclusions to the paper.

## 2. Materials and Methods

**Figure 1:**
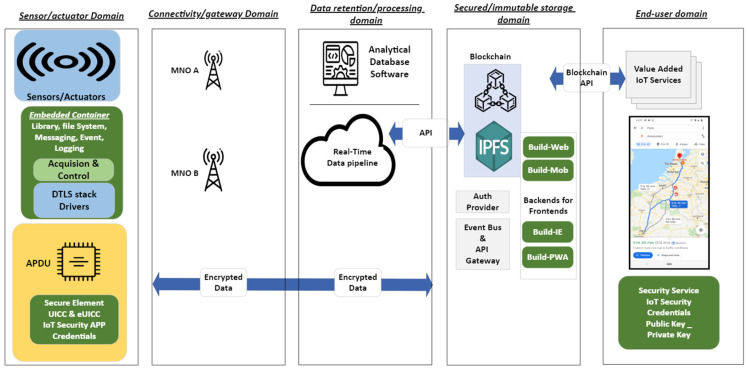
Architecture for MIoT with distinct domains.

A five-layer architectural framework has been designed and implemented to accommodate the demands of the MIoT platform, as shown in Figure 1. This design facilitates connectivity for a wide range of IoT devices such as sensors and actuators, demonstrating a profound efficiency. In order to reach an efficient platform, we tried to emphasize retention, processing, and secured storage capabilities in this architecture, which, respectively, are provided by Apache Kafka, Apache Druid, and blockchain technology.

### 2.1. Domains and Data Flow

Recognizing the need to tackle diverse challenges, we opted to structure the model into five distinct domains. This approach was chosen to optimize our solution. The initial domain, comprising sensing and actuating devices, plays the role of data acquisition. To ensure availability, domain 2 (connectivity and gateway) employs dual mobile network operators (MNOs) and automatic switchover, thereby safeguarding framework availability in case of failure. As mentioned earlier, we use two LTE-based SDNs. The subsequent domain not only delivers scalability, real-time data processing, and minimal latency but also incorporates data retention capabilities. Storage security measures are established within the fourth domain. A concise overview of these domains and their respective functionalities is provided in Table 1.

**Figure 2:**
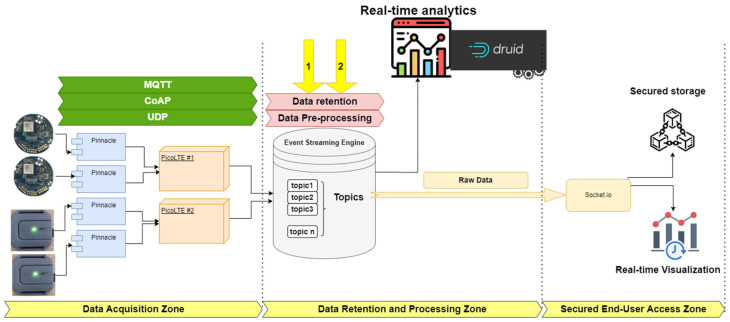
Data flow featured with three zones and two phases in the middle zone: 1. Data retention 2. Data pre-processing.

To illustrate the data flow intricacies, a three-zone framework was employed to depict the entire process, from data collection to its secure storage on the blockchain. The process initiates with data collection by sensors, followed by its transmission to gateway devices and subsequently network simulators. The network simulators, represented by our simulated dual SDRs, namely Pico LTE acting as a Network-in-a-Box, establish connectivity to Kafka. Employing protocols outlined in Figure 2, they function as publishers or producers, facilitating data transfer to Kafka. Meanwhile, Hyperledger Fabric subscribes to specified Kafka topics via socket.io, ensuring secure access for end-users. Additionally, Apache Druid, functioning as a real-time data analytics tool, serves as another consumer in the chain.

#### 2.1.1. Sensor and Actuator Domain

**Figure 3:**
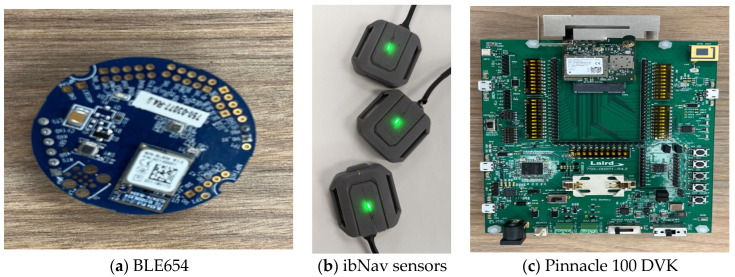
Devices used as sensors and gateways: (**a**) BL654; (**b**) ibNav sensors; (**c**) Pinnacle 100 DVK.

As implied by the domain’s name, it hosts a variety of devices such as sensors, actuators, and board computers for capturing environmental data. For our study, we utilized BL654 and ibNav sensors to execute data collection. Pinnacle^TM^ 100 DVK devices from Laird Connectivity manufacturing company in Akron, OH, USA, were adopted as gateway elements which were equipped with a SIM card for connectivity purposes. Notably, we harnessed Wi-Fi for ibNav and Bluetooth for Pinnacle to facilitate data transmission from sensors to gateways. This, in turn, enabled onward transmission to the network layer, housing two SDR devices from Nutaq. Leveraging BL654 sensors, we effectively captured temperature, humidity, and pressure readings. With ibNav, our scope expanded to encompass position data alongside the aforementioned metrics. The devices in use are depicted in Figure 3.

#### 2.1.2. Gateway and Connectivity Domain

With the inclusion of network simulators, we can emulate LTE-M, an optimal connectivity protocol, to achieve fast, high-rate data transfer with minimal latency [19]. The network domain ensures the smooth transition of data from the sensor/actuator domain to the storage and processing domain.

**Figure 4:**
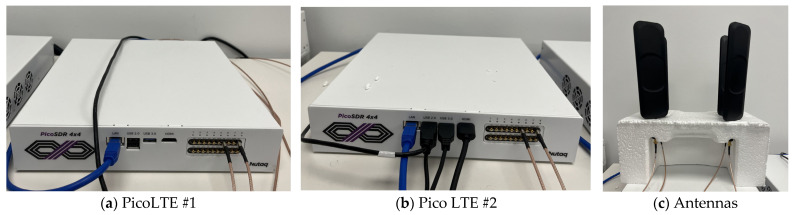
Two PicoLTEs and their antennas.

Our experimental configuration incorporated two Nutaq PicoLTE second-generation devices, skillfully simulating two mobile network operators (MNOs), as shown in Figure 4. Leveraging the capabilities of PicoLTE, we established a dynamic and robust simulation platform. This enabled the creation of an LTE-centric network environment for our MIoT experiments and streamlined the assessment of our MIoT system’s performance and behavior.

Nutaq’s first-generation PicoLTE showcases a range of attributes, making it a compelling solution for various LTE needs. Characterized by its compact and portable design, this all-in-one integrated solution offers support for LTE Cat 0, Cat 1, Cat M1, Cat NB1, and Cat NB2, ensuring compatibility across multiple LTE generations. The cost-effectiveness and affordability of this solution stands out, providing an accessible option for diverse deployments. Configuration support is offered for all LTE bands, encompassing both the Frequency Division Duplex (FDD) and Time Division Duplex (TDD) modes.

#### 2.1.3. Data Retention and Processing Domain

Within our envisioned model, this specific domain assumes the critical role of preserving and performing real-time analytics on the massive amount of data generated by IoT devices. By utilizing data retention regulations in Apache Kafka, we achieve the dual advantage of customized data storage duration and the capability to execute various data manipulations. These manipulations encompass aggregation, filtering, data-type transformation, field redaction, etc.

Simultaneously, Apache Druid facilitates real-time data processing, enhancing the framework’s capacity for instantaneous analytics. In data analytics, the choice between a data warehouse and a real-time analytics database hinges on the task at hand. Snowflake, a notable example of a data warehouse, excels at reporting and data consolidation. Its cost-efficient architecture prioritizes low-cost storage and allows for concurrency through clustering, making it ideal for periodic reports and insights. In contrast, Apache Druid represents the domain of real-time analytics databases, specifically designed for interactive data conversations with sub-second response times, high concurrency, and flexibility. Moreover, Druid’s dynamic schema approach adapts to changing data, while its focus on low-latency querying and real-time analytics makes it a powerful solution for swiftly processing large volumes of streaming data. Table 2 provides a comparison in this regard.

#### 2.1.4. Secured/Immutable Storage Domain

In this domain, the blockchain and database collaborate to establish a decentralized and secure ledger. This partnership guarantees data integrity and immutability by preserving hashed versions of the transmitted data. Serving as a conduit, Kafka receives data from devices and conveys it through the pipeline to the database. Following this, the data undergo a secure hashing process, and the resultant hash is archived within the blockchain, creating an immutable history of the initial data.

In our research, we incorporated the Inter Planetary File System (IPFS) to assist the blockchain. This integration facilitates the hosting of produced MIoT data, improving the overall system’s performance. IPFS serves as a valuable complement to blockchain since it functions as a decentralized peer-to-peer network for file sharing and storage, relying on unique resource addresses to locate and retrieve data through Distributed Hash Tables (DHTs). The IPFS efficiently minimizes redundancies and conserves storage space using file hashes. It is essential to note that IPFS operates differently from blockchain. Once a file is uploaded to the network, it is impossible to be removed intentionally. However, IPFS necessitates periodic cache clearing, gradually phasing out less popular files from the network [20].

#### 2.1.5. End-User Domain

Within IoT ecosystems, the end-user domain delivers significant value to its designated users. This domain offers a user-friendly interface for users that allows them to log in and to monitor their IoT devices. In other words, in a smart device ecosystem, this domain is responsible for analyzing sensor data, generating visual representations of device performance, and providing a user interface for initiating firmware upgrades. These upgrades ensure that devices remain equipped with the latest features, security enhancements, and performance improvements. Users can effortlessly initiate firmware updates through the application interface, ensuring device maintenance and feature enhancement. Consequently, the end-user domain in the IoT ecosystem serves the dual purpose of data visualization and firmware management.

User authentication within this domain is paramount to enable communication with the blockchain. To ensure secure access, a two-layer authentication mechanism is employed. The first layer handles the initial user authentication, while the second layer leverages the blockchain’s inherent authentication procedures. Both layers work in tandem to verify the user’s identity. The data presented to the user are based on their specific needs and access credentials. This layer acts as the gateway for user interaction with the blockchain, enabling users to submit requests and to receive responses. Additionally, this layer offers the flexibility to develop other applications, specified for individual user requirements [19].

### 2.2. Publish/Subscribe Method for Data Retention and Processing

The publish/subscribe messaging approach is characterized by the sender (publisher) of the data which is not explicitly targeting a particular recipient. Instead, the publisher categorizes the message in some manner, and the recipient (subscriber) subscribes to receive specific categories of messages. Typically, publish/subscribe systems employ a broker, a central hub where messages are published [21].

**Figure 5:**
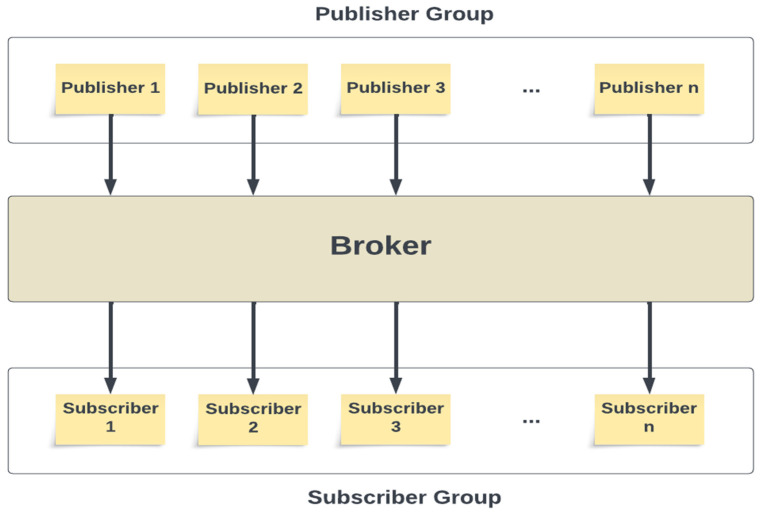
The logic behind publish/subscribe system architecture.

Figure 5 depicts the logic behind the publish/subscribe system architecture. Within this system, numerous producers and consumers are at play. Each producer corresponds to an application or device responsible for generating diverse data types—such as temperature, humidity, pressure, position tracking data, etc.—which are then transmitted to the central *broker*. On the other side, consumers have the flexibility to asynchronously subscribe to specific data types as needed. This approach greatly surpasses the traditional request/response or point-to-point methods. To effectively organize and manage messages within the broker, a system of *topics* is employed, and these *topics* are further divided into *partitions*.

#### Apache Kafka as Stream Processing Platform

Apache Kafka, a platform which uses the publish/subscribe method, emerged as a solution to the challenges posed by traditional request/response messaging systems. A database commit log in Kafka serves the purpose of maintaining customized durations so that the data can be read consistently. Furthermore, Kafka’s data can be distributed across the system, offering increased safeguards against failures (availability) and substantial potential for performance improvement.

**Figure 6:**
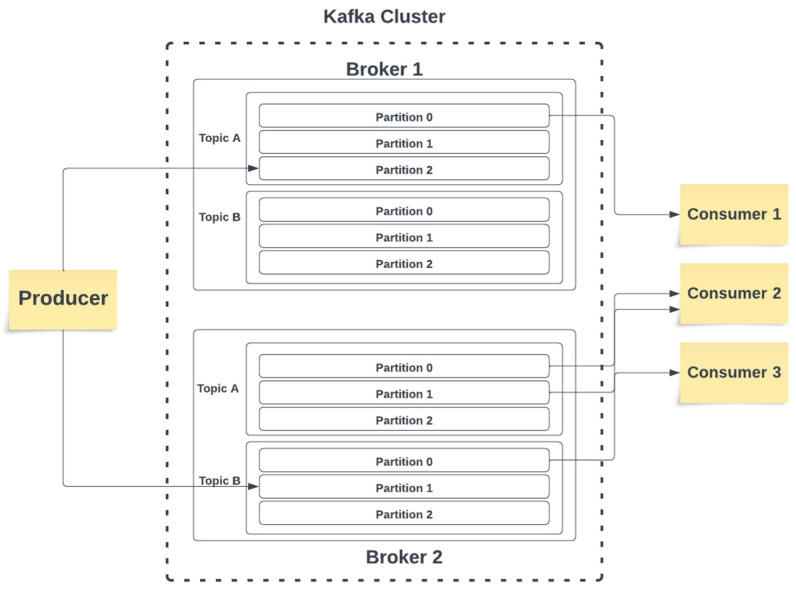
Apache Kafka fundamental components.

The current state of Apache Kafka is undeniably exciting. Kafka has been adopted by numerous organizations all around the world. This open-source platform has rapidly ascended as one of the fastest-growing projects in the open-source community [22]. At its core, Kafka drives the trend of efficiently handling and analyzing data streams. Illustrated in Figure 6, Apache Kafka comprises fundamental components, namely *producer*, *cluster*, *broker*, *topic*, *partition*, and *consumer*.

- **Broker**: Within Kafka, a multitude of brokers exist in the ecosystem. Their primary responsibility is to accept messages from producers and to subsequently commit them to a designated Kafka topic.
- **Producer**: Producers are responsible for creating new messages and delivering them to a Kafka topic in a specific broker.
- **Consumer**: Consumers subscribe to one topic or sometimes multiple topics and read messages. Since consumers store the tracking history of the consumed data, they can pause and resume their consumption progress without losing their position.
- **Cluster**: In the Kafka ecosystem, clusters can house multiple brokers within them. Within each cluster, there exists a broker tasked with the crucial duty of monitoring broker failure. This broker is called the *controller*.

- **Topic**: In the Kafka system, messages are neatly categorized into topics, functioning much like database tables. These topics play the role of maintaining data, ensuring its retention over a pre-defined time frame. Each broker has the capacity to host one or multiple topics.
- **Partition**: Within each topic, multiple partitions serve as containers for logging messages. When messages are sent to these topics, they are written to partitions using an append-only approach, signifying that the data remain unaltered and immutable during the reading process.

The data generated by IoT devices, serving as producers, is initially routed to Kafka brokers. Within the broker infrastructure, the data are directed to a queue. Subsequently, the broker ensures the data’s persistence by allocating them to a specific partition within a designated topic. On the consumer side, which, in our project, is represented by the blockchain component, subscriptions are made to the broker’s partition or topic, and data are consumed from the queue asynchronously. Vital metadata, indicating which data have already been processed and which remain, are maintained in Kafka Zookeeper. Any necessary actions are relayed back to the broker, subsequently reaching the IoT devices. In this context, the blockchain serves as the producer, while IoT devices function as consumers in our system. Latency is measured using the equation below:

*T*_total_ = *T*_DB_ + *W*_TDB_ + *T*_B_ + *T*_BC_ + *W*_TBC_ + *R*_TBC_ + *R*_TBD_ (1)

This equation accounts for the various time components involved in the data processing latency, including the time for publishing data by IoT devices (*T*_DB_), the wait time in the queue for data processing at the broker (*W*_TDB_), the processing time in the broker layer (*T*_B_), the time required to transfer the data processed by the broker to the blockchain component (*T*_BC_), the wait time in the queue for data processing at the blockchain component (*W*_TBC_), the time required to transfer the processed data back to the broker by the blockchain component (*R*_TBC_), and the time required to transfer the information back to the IoT devices through the broker (*R*_TBD_). The latency metrics observed within the queue are detailed in the corresponding Table 3.

## 3. Results and Analysis

The research presented in this article focused on addressing the challenges of data processing and communication within MIoT using the publish/subscribe method and leveraging blockchain for secured data storage, with a specific emphasis on latency. Throughout the experimentation and analysis, several key findings regarding latency were observed, shedding light on the system’s performance and effectiveness. With reference to latency measuring, we employed Kafka’s Confluent Center, as well as Apache Druid, a built-in feature for showing statistics along with predefined use cases. In terms of hardware, we utilized a Ubuntu machine with the configurations detailed in Table 4.

### 3.1. Latency of Apache Druid without Apache Kafka

**Figure 7:**
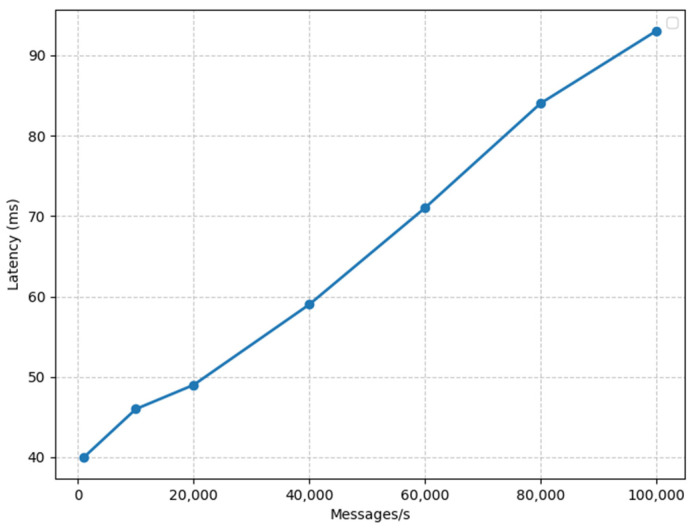
Apache Druid’s latency without Kafka.

In Figure 7, we present the latency measurements for Apache Druid in an environment where Apache Kafka is not in operation. The graph illustrates how latency changes under varying message rates. Notably, we observe a significant increase in latency when Apache Druid is subjected to a message rate of 100,000 messages per second. It is essential to note that the messages in this context consist of temperature, humidity, and pressure values, which are not typically high in volume.

### 3.2. Latency with Apache Kafka in Operation

**Figure 8:**
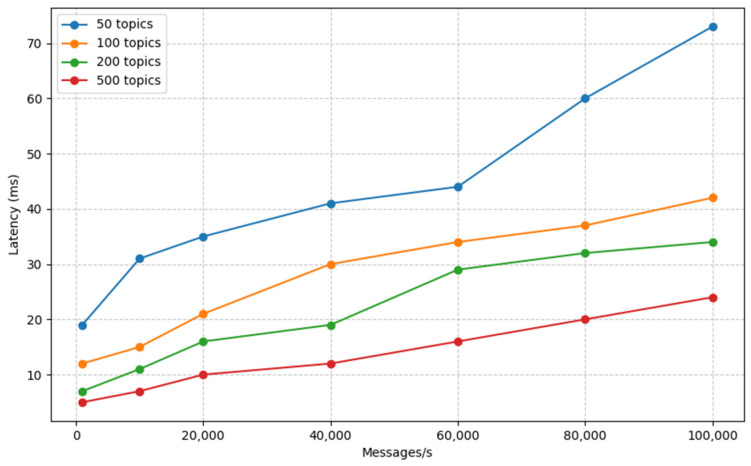
Apache Druid latency with Kafka.

In Figure 8, we explore the impact of incorporating Apache Kafka with different numbers of topics in a single-broker setup. Here, we observe that the latency remains consistently low, reaching less than 25 milliseconds even when processing 100,000 messages per second. When compared to [5], their results reveal an average latency of approximately 25 ms for a workload of 60 messages per second for a message size of 10,000 KB. It is noteworthy that their scale may not be indicative of MIoT, emphasizing the distinction in the performance evaluation between the two studies. This fact demonstrates the efficiency and reliability of our utilization of Kafka to handle data streams with diverse topics, ensuring minimal latency for real-time data processing.

To sum up, our systems are designed to efficiently manage up to 100,000 sensors, each capable of transmitting low-volume measurements every second, when accommodating 500 topics. All of these conditions apply when we are operating within a framework of consistently low latency.

### 3.3. Request Pool Usage

**Figure 9:**
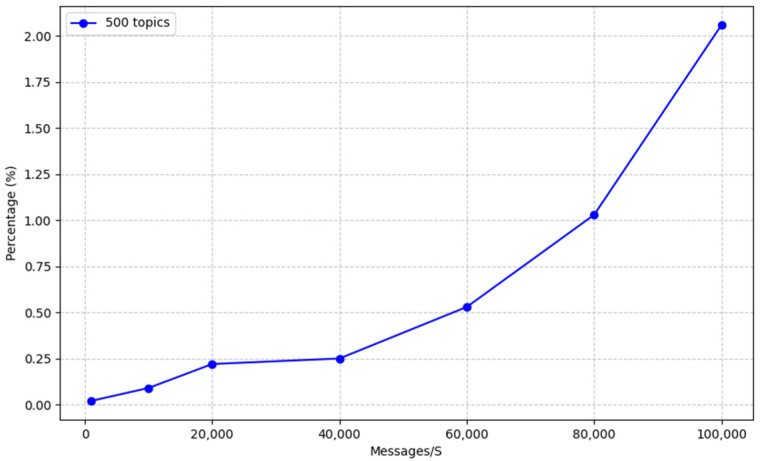
Request pool usage.

As previously discussed, each broker features a dedicated queue. When a producer dispatches a message, it enters the queue, where it predominantly remains in an idle state, occasionally transitioning to an active state. Request pool usage represents the average capacity utilization of request handlers across all brokers. In other words, it quantifies the percentage of time that the request handler threads are actively engaged and not in an idle state. Figure 9 illustrates that, at a data rate of 100,000 messages per second, the request handler is active for just 2.06 percent of the time, highlighting an exceptionally efficient utilization of resources.

### 3.4. Throughput and Availability Trade-off: One Broker vs. Two Brokers

**Figure 10:**
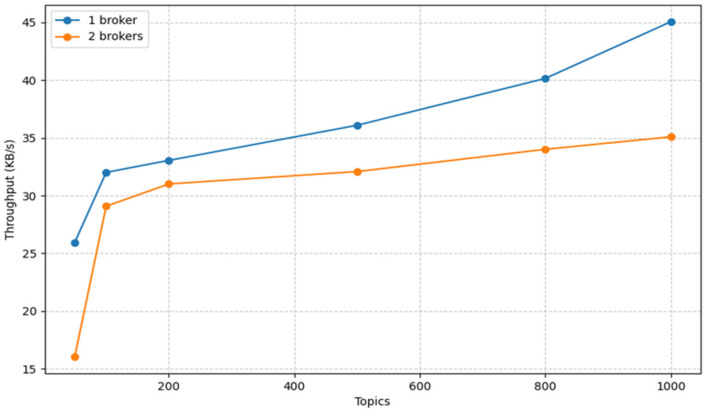
Throughput comparison with one and two brokers.

In this section, we explore the crucial trade-off between throughput and availability in our system. Our investigation uncovers an intriguing dynamic: a single-broker configuration yields superior throughput, while introducing two brokers may result in a slight degradation. In a dual-broker configuration, one broker assumes the role of the *leader* while the other acts as the *follower*. This setup ensures the replication of partitions from the leader broker to the follower broker in the event of a broker failure, guaranteeing continuous data availability. Notably, with the introduction of two brokers, there is a marginal, albeit not substantial, decrease in throughput, as depicted in Figure 10.

### 3.5. Blockchain Performance

**Figure 11:**
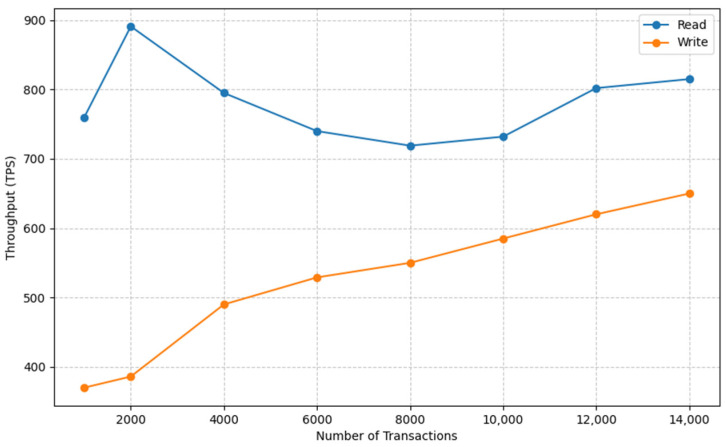
Performance of Hyperledger.

We utilized Hyperledger Caliper, a freely available open-source tool, to assess the performance of our blockchain platform. Our testing involved Hyperledger Fabric utilizing hardware identical to that of the Kafka test. In Figure 11, we present the performance results for writing and reading transactions during our evaluation. The figure vividly demonstrates a positive correlation between Transactions Per Second (TPS) and the number of transactions, underscoring the efficiency of the proposed platform.

Furthermore, our findings, as depicted in Figure 11, reveal that as the transaction count escalates, the success rate of accepting read transactions remains consistently high. This observation underscores the network’s robust capability to handle massive read requests while maintaining the accuracy and reliability of the data retrieval process.

## 4. Discussion

In this section, we will elaborate on positive aspects associated with adopting the proposed MIoT framework which empowers enterprises to access a variety of valuable results and to elevate their overall functioning. In this evolving landscape of the Internet of Things, our paper champions the use of the publish/subscribe approach, integrating Apache Druid and Apache Kafka for enhanced scalability and low latency in MIoT applications, which reaches less than 25 ms for 100,000 sensors in contrast to existing works that report 25 ms latency for significantly fewer devices. Supporting such a number of sensors, bolstered by dual mobile network operators for connectivity resilience and, on the other hand, utilizing Hyperledger Fabric for storage security, for which its performance remained high with more than 800 successful TPS in 14,000 transactions, our model signifies a breakthrough in MIoT innovation, opening avenues for future research and advancements. The positive aspects can be divided into six sections, as follows:

- **Low latency:** Utilizing Kafka’s publish/subscribe architecture eliminates the need for real-time app control and data allocation since data are available for subscription from any IoT application in Kafka topics asynchronously. This attribute significantly contributes to our framework’s low-latency performance.
- **Data retention with Kafka:** Kafka’s data retention feature is a crucial asset in our framework. It guarantees that data remain accessible even in situations where latency or disruptions may occur. By retaining data over extended periods, Kafka ensures the reliability and resilience of the system, enabling historical data analysis and auditing capabilities.
- **Scalability:** The framework’s scalability stems from Kafka’s ability to deploy brokers across diverse clusters within various cloud services. Indeed, partitioning is crucial because when a partition is scaled in multiple brokers, various consumers can concurrently consume from the same partition across diverse clusters, particularly beneficial during high message traffic periods for a given Kafka topic.
- **Availability:** Our framework places a significant emphasis on ensuring uninterrupted service availability. Kafka’s inherent fault tolerance, achieved through data replication and distributed architecture, plays a pivotal role in this regard. Additionally, the introduction of multiple brokers and leader–follower configurations, as discussed earlier, contributes to high availability, as it guarantees data continuity even in the event of a broker failure. Last but not least, having two MNOs guarantees connectivity availability in case of connection failure.
- **Integrity and security:** Leveraging blockchain technology, our framework secures data with a high commitment to integrity. Blockchain’s inherent immutability ensures that data remain tamper-proof, maintaining the trustworthiness of stored information. Robust security measures, including multi-layered authentication protocols and encryption, further fortify our system against potential threats, providing a shield for the protection of sensitive data.
- **Monitoring and insights:** Administrators can harness our framework to gain profound insights from the massive data flow. With the ability to monitor data in real time and to conduct in-depth analyses, administrators can make informed decisions, identify trends, and proactively address issues, enhancing the overall efficiency of IoT operations.

In summary, our study not only combines advanced technologies effectively to address challenges in the realm of MIoT but also opens avenues for further research and innovation. While the publish/subscribe method proves robust for many scenarios, it is important to note its limitations, particularly in synchronous communication scenarios such as media streaming. However, by integrating blockchain, Kafka, and robust security measures, we have constructed a framework that excels in terms of low latency, scalability, availability, data integrity, and data retention, making it a powerful solution for various IoT applications.

## 5. Conclusions

As the landscape of the Internet of Things continues to evolve, the proliferation of Machine-Type Devices (MTDs) has given birth to the concept of MIoT, a significant trend in the realm of connected devices. In this paper, we have championed the use of the publish/subscribe approach for data storage, delivering notable improvements in scalability and latency for the interconnected world of IoT. By incorporating Apache Druid, an analytical database software, into our framework, we have empowered real-time data processing, further enhancing the capabilities of MIoT applications. Our strategic choice of Apache Kafka as the data transmission platform has streamlined the flow of data from sensors to brokers, creating an efficient subscription ecosystem that caters to the dynamic needs of IoT applications. With our proposed architecture, we have projected that the system could effectively support an IoT network comprising a minimum of 100,000 sensors, each transmitting low-volume messages per second, across a network featuring 1 broker and 500 topics.

Moreover, we have bolstered the resilience of our connectivity by simulating the presence of two mobile network operators (MNOs) through Software-Defined Radio (SDR) devices, providing a reliable backup for uninterrupted data transmission, even in the face of connection failures.

Notably, our commitment to data security is unwavering, exemplified by our deployment of Hyperledger Fabric, a cutting-edge blockchain technology celebrated for its robust security and data integrity features. The results of our comprehensive analysis underscore the model’s core attributes, highlighting its remarkable scalability, unwavering availability, robust security protocols, and minimal latency, positioning it as a formidable solution in the ever-evolving area of MIoT.

Furthermore, it is essential to recognize that while the publish/subscribe approach proves to be robust for numerous MIoT scenarios, its effectiveness in synchronous communication scenarios, such as media streaming, may have limitations. These aspects, among others, open doors for future research and innovation, inviting further exploration into the dynamic and multifaceted world of MIoT. As the MIoT ecosystem continues to expand and diversify, our approach not only pushes the boundaries of what is currently achievable but also sets a new standard for the future of connected devices and data processing. This paves the way for ongoing advancements and innovations in the exciting field of MIoT, providing a solid foundation for building smarter, more responsive, and highly efficient IoT applications that cater to the diverse needs of our connected world.

## Figures and Tables

**Table 1:** Brief description of the domains.

Domains	Description
1st Domain—Sensor/actuator	Includes devices to collect various data, e.g., temperature, humidity, pressure, position, etc.
2nd Domain—Connectivity and gateway	Includes Pico LTE, which is an SDR to simulate MNOs in order to provide various connectivity protocol, e.g., LTE Cat 0, Cat 1, Cat M1, Cat NB1, and Cat NB2
3rd Domain—Data retention and processing	Includes Apache Kafka and Apache Druid for data retention and real-time processing
4th Domain—Secured/immutable storage	Includes Hyperledger Fabric to supply with storage security
5th Domain—End-user	Includes smart home application in order to manage and track IoT devices

**Table 2:** Summary of Apache Druid advantages vs. data warehouse.

Data Warehouse	Apache Druid as a Real-Time Analytic Tool
Provides reporting without considering performance	Provides reporting while performance is important (interactive data conversation)
Focuses on low cost	Focuses on low latency (fast)
Cluster concurrency	High concurrency
Inflexible, locking (fixed schema)	Flexible in heterogenous data types (fixed schema, flexible schema)

**Table 3:** Broker’s queue properties and description.

Queue Properties	Description
Response Send	Measures the time taken by the broker to send a response to the client after receiving a request
Request Queue	Measures the time a client request spent waiting in the broker’s request queue to be processed
Request Local	Measures the time taken by the broker to process a client request that was directed to the same broker
Response Remote	Measures the time taken by the broker to receive a response from another broker in the Kafka cluster
Response Queue	Measures the time a response spent waiting in the broker’s response queue to be sent back to the client
Median	Represents the 50th percentile of the distribution of the response time for requests in this broker

**Table 4:** Simulation parameters.

Parameters	Value
CPU	Intel(R) Core(TM) i7-9750U CPU @ 2.60 GHz
RAM	16 GB
Storage	512 GB
No. of Kafka clusters	1
No. of Kafka brokers	1 and 2
No. of Kafka topics	50 to 500
No. of messages per second	1000 to 100,000

## Data Availability

Due to confidentiality issues, we are not able to share data.
